# The Runx transcriptional co-activator, CBFβ, is essential for invasion of breast cancer cells

**DOI:** 10.1186/1476-4598-9-171

**Published:** 2010-06-30

**Authors:** Daniel Mendoza-Villanueva, Wensheng Deng, Cesar Lopez-Camacho, Paul Shore

**Affiliations:** 1 Faculty of Life Sciences, University of Manchester, Michael Smith Building, Oxford Road, Manchester, M13 9PT, UK

## Abstract

**Background:**

The transcription factor Runx2 has an established role in cancers that metastasize to bone. In metastatic breast cancer cells Runx2 is overexpressed and contributes to the invasive capacity of the cells by regulating the expression of several invasion genes. CBFβ is a transcriptional co-activator that is recruited to promoters by Runx transcription factors and there is considerable evidence that CBFβ is essential for the function of Runx factors. However, overexpression of Runx1 can partially rescue the lethal phenotype in CBFβ-deficient mice, indicating that increased levels of Runx factors can, in some situations, overcome the requirement for CBFβ. Since Runx2 is overexpressed in metastatic breast cancer cells, and there are no reports of CBFβ expression in breast cells, we sought to determine whether Runx2 function in these cells was dependent on CBFβ. Such an interaction might represent a viable target for therapeutic intervention to inhibit bone metastasis.

**Results:**

We show that CBFβ is expressed in the metastatic breast cancer cells, MDA-MB-231, and that it associates with Runx2. Matrigel invasion assays and RNA interference were used to demonstrate that CBFβ contributes to the invasive capacity of these cells. Subsequent analysis of Runx2 target genes in MDA-MB-231 cells revealed that CBFβ is essential for the expression of Osteopontin, Matrixmetalloproteinase-13, Matrixmetalloproteinase-9, and Osteocalcin but not for Galectin-3. Chromatin immunoprecipitation analysis showed that CBFβ is recruited to both the Osteopontin and the Galectin-3 promoters.

**Conclusions:**

CBFβ is expressed in metastatic breast cancer cells and is essential for cell invasion. CBFβ is required for expression of several Runx2-target genes known to be involved in cell invasion. However, whilst CBFβ is essential for invasion, not all Runx2-target genes require CBFβ. We conclude that CBFβ is required for a subset of Runx2-target genes that are sufficient to maintain the invasive phenotype of the cells. These findings suggest that the interaction between Runx2 and CBFβ might represent a viable target for therapeutic intervention to inhibit bone metastasis.

## Background

CBFβ is a transcriptional co-activator that is recruited to promoters by members of the Runx family of transcription factors. Runx transcription factors are defined by the presence of a conserved DNA-binding domain, termed the Runt domain, that recognises the consensus sequence ACC(A/G)CA [1]. The Runt domain also interacts with CBFβ. CBFβ binds to the non-DNA-binding surface of the Runt domain to induce structural changes in the DNA-recognition surface, thereby increasing its affinity for DNA [2,3]. CBFβ is essential for haematopoiesis and the development of the skeleton, by virtue of its interaction with Runx proteins [4-6]. Indeed, CBFβ is essential for most of the known functions of Runx proteins. However, there is evidence that in some situations Runx proteins can regulate gene expression independently of CBFβ. In the sea urchin, CBFβ is not required for expression of the Runx target gene PKC1 [7,8]. Moreover, overexpression of Runx1 partially rescued the lethal phenotype in CBFβ-deficient mice, indicating that overexpressed Runx1 can regulate gene expression in the absence of CBFβ [9].

Runx2 is overexpressed in breast cancer cell lines that metastasize to bone where it has an established role in invasion. When Runx2 function was inhibited in metastatic breast cancer cells transplanted to bone, tumorigenesis and osteolysis were prevented [10]. Runx2 regulates the expression of several genes known to be involved in cell migration and metastasis including, Matrixmetalloproteinase-13 (MMP-13) and Matrixmetalloproteinase-9 (MMP-9), Vascular Endothelial Growth Factor (VEGF) and Bone Sialoprotein (BSP)[11-13]. Ablation of Runx2 expression in metastatic breast cancer cells, MDA-MB-231, resulted in down-regulation of metastatic genes and reduced the invasive capacity of the cells [12]. However, it is not known if the increased expression of Runx2 observed in metastatic breast cancer cells is sufficient to regulate gene expression independently of CBFβ. Indeed, it is not known if CBFβ is expressed in metastatic breast cancer cells.

Here we demonstrate that CBFβ is expressed in metastatic breast cancer cells and that it is essential for cell invasion. We also show that several Runx2-target genes, known to be involved in cell invasion, require CBFβ. However, whilst CBFβ is essential for invasion not all Runx2 target genes require CBFβ. We conclude that CBFβ is required for a subset of Runx2-target genes that are sufficient to maintain the invasive phenotype of the cells. These findings suggest that the interaction between Runx2 and CBFβ might represent a viable target for therapeutic intervention to inhibit bone metastasis.

## Results

### CBFβ is associated with Runx2 in the metastatic breast cancer cell line MDA-MB-231

**Figure 1:**
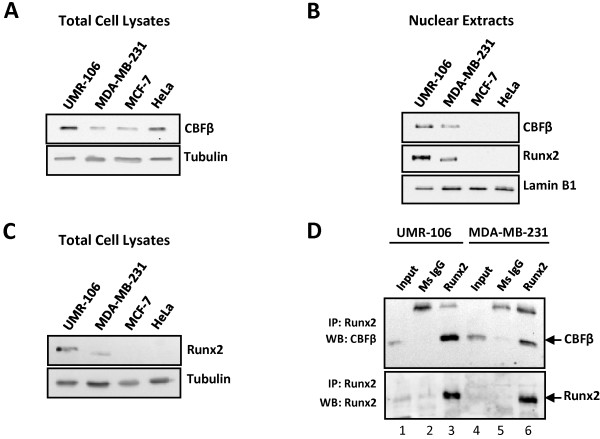
**CBFβ is associated with Runx2 in the metastatic breast cancer cell line MDA-MB-231**. (A) Western blot showing CBFβ expression in MDA-MB-231 cells. Total extracts from metastatic (MDA-MB-231) and non-metastatic (MCF-7) breast cancer cell lines were subjected to western blotting analysis with a CBFβ antibody. HeLa cells known to express CBFβ were used as positive controls. Protein levels of Tubulin are shown as an internal loading control. (B) CBFβ and Runx2 are nuclear in MD-MB-231 cells. Nuclear extracts from MDA-MB-231 and MCF-7 cells were utilized to determine the localisation of Runx2 and CBFβ. Lamin B1 was used as loading control. (C) Western blot showing Runx2 expression in MDA-MB-231 cells. Total extracts from metastatic (MDA-MB-231) and non-metastatic (MCF-7) breast cancer cell lines were subjected to western blotting analysis with a Runx2 antibody. The UMR-106 and HeLa cells were used as positive and negative controls for Runx2 expression, respectively. Protein levels of Tubulin are shown as an internal loading control. (D) Runx2 interacts with CBFβ in MDA-MB-231 cells. Whole extracts of the UMR-106 and MDA-MB-231 cells were subject to immunoprecipitation (IP) assays. A monoclonal antibody against Runx2 was used to immunoprecipitate the Runx2 protein. Then, immunoblots with a polyclonal antibody anti-CBFβ (upper) or a whole serum anti-Runx2 (lower) were used to determine the presence of CBFb and Runx2, respectively. IgG antibody was used as a specificity control. Whole extracts of the cells (input) were used as control for CBFβ and Runx2 expression.

Previous reports have shown that compared to non-metastatic cells Runx2 is overexpressed in the metastatic breast cancer cells, MDA-MB-231 [13,14]. Overexpression of Runx1 is known to be sufficient to rescue the lethal phenotype in CBFβ-deficient mice, indicating that increased levels of Runx factors can at least partially overcome the requirement for CBFβ [9]. To determine whether CBFβ has a role in the invasive capacity of metastatic MDA-MB-231 cells we first established that it is expressed in these cells. Immunoblotting of total cell extracts with an anti-CBFβ antibody demonstrated that CBFβ is present in both MDA-MB-231 and the non-metastatic MCF-7 cells (Fig. 1A). CBFβ was also observed in the osteosarcoma cell line, UMR-106, and HeLa cells (Fig. 1A). CBFβ is therefore expressed in both metastatic and non-metastatic breast cancer cell lines. However, in nuclear extracts, CBFβ was present in MDA-MB-231 and UMR-106 cells but not in MCF-7 and HeLa cells (Fig. 1B). In agreement with previous reports, western blotting demonstrated that Runx2 is expressed in the metastatic MDA-MB-231 cells but was not detectable in non-metastatic MCF-7 cells (Figs. 1B &1C). Runx2 is expressed in UMR-106 cells but not in HeLa cells, which were used as positive and negative controls respectively. Thus, CBFβ is present in the nucleus of cells expressing Runx2.

To establish if CBFβ is associated with Runx2 in MDA-MB-231 cells, endogenous Runx2 was immunoprecipitated with an anti-Runx2 antibody followed by western blotting with an anti-CBFβ antibody (Fig. 1D). CBFβ co-precipitated with Runx2 in both MDA-MB-231 and UMR-106 (Fig. 1D, lanes 3 & 6). The association was specific, as CBFβ did not co-precipitate with a non-specific IgG antibody (Fig. 1D, lanes 2 & 5). These data demonstrate that the Runx2/CBFβ complex is present in MDA-MB-231 cells.

### Depletion of CBFβ in MDA-MB-231 cells reduces their invasive capacity

**Figure 2:**
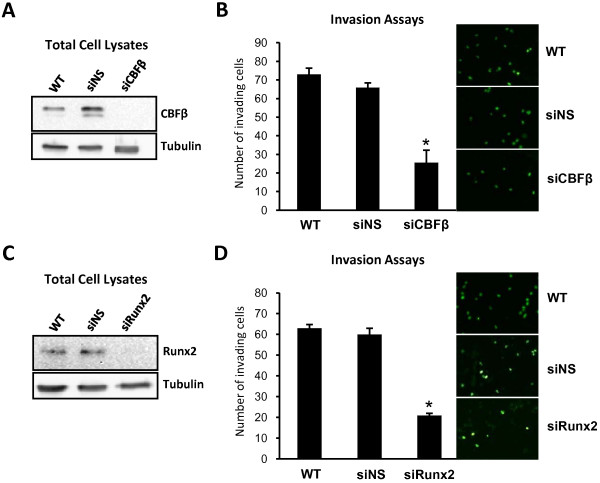
**siRNA-mediated depletion of CBFβ in MDA-MB-231 cells inhibits their invasive capacity**. (A) Western blot showing siRNA knockdown of CBFβ. The upper panel shows total cell lysates derived from siRNA transfections of MDA-MB-231 cells after immunodetection with an anti-CBFβ antibody. The lower panel is a Tubulin loading control. (B) MDA-MB-231 cells were transfected with siRNA against CBFβ (siCBFβ) and a non-specific siRNA (siNS). 24 hrs after a second transfection the cells were plated on Matrigel for invasion assays. Wild type (WT) MDA-MB-231 cells were used as positive control for invasion. Cells that migrated were stained and counted in random fields. Data are presented as mean ± standard deviation (S.D.) (n = 3). * indicates p < 0.05 compared with siNS by analysis of variance. (C) Western blot showing siRNA knockdown of Runx2. The upper panel shows total cell lysates derived from siRNA transfections of MDA-MB-231 cells after immunodetection with an anti-Runx2 antibody. The lower panel is a Tubulin loading control. (D) Runx2 is required for invasion of MDA-MB-231 cells. MDA-MB-231 cells were transfected with siRNA against Runx2 (siRunx2) or a non-specific siRNA (siNS). 24 hrs after a second transfection the cells were plated on Matrigel for invasion assays. Wild type (WT) MDA-MB-231 cells were used as a positive control for invasion. Cells that migrated were stained and counted in random fields. Data are presented as mean ± standard deviation (S.D.) (n = 3). * indicates p < 0.05 compared with siNS by analysis of variance.

Having shown that MDA-MB-231 cells express CBFβ we next sought to determine whether CBFβ contributed to their invasive capacity. siRNAs were therefore used to knock down CBFβ by transient transfection of MD-MB-231 cells. Matrigel invasion assays were subsequently performed to determine the migratory capacity of the cells. CBFβ protein was reduced by over 90% in cells transfected with siRNAs targeted against CBFβ, but was unaffected in non-specific siRNA control transfections (Fig. 2A). Cells with reduced CBFβ expression exhibited significantly less invasion (34%) than control cells (90%) when compared with wild-type MDA-MB-231 cells (100%) (Fig. 2B). Decreased expression of CBFβ therefore significantly reduces the invasive capacity of MDA-MB-231 cells. These findings mirrored the effect of knocking down Runx2 which, in agreement with a previous report, also significantly inhibited the invasion capacity of MDA-MB-231 cells (Figs. 2C &2D)[12].

**Figure 3:**
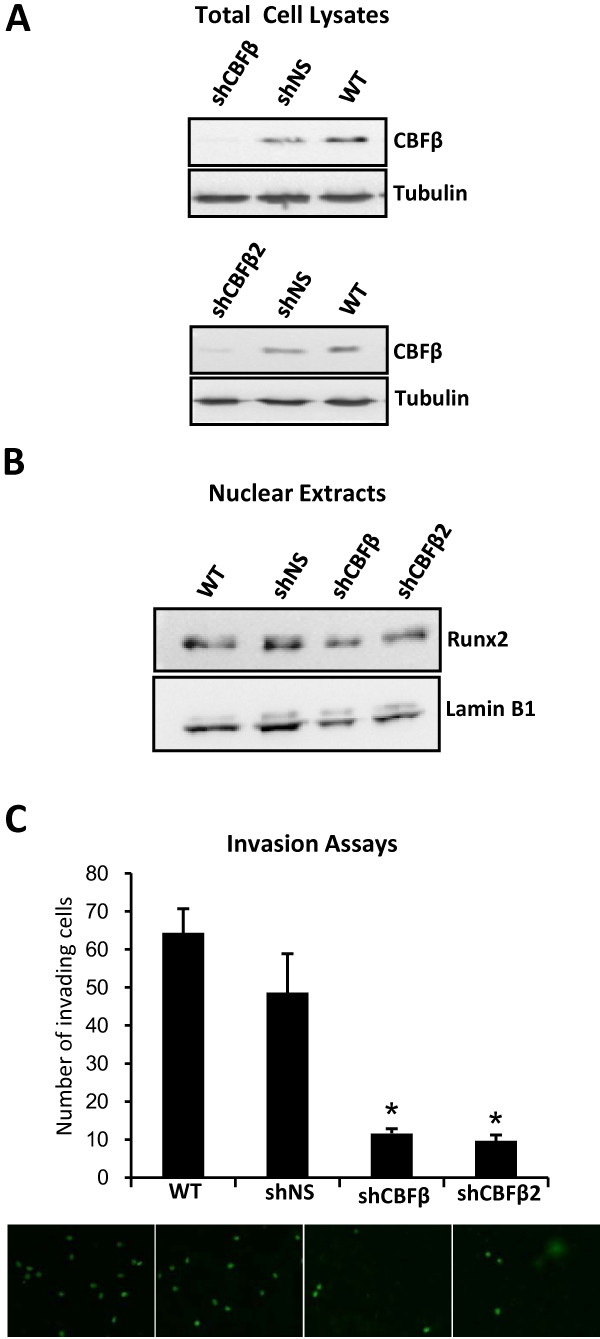
**Stable knockdown of CBFβ inhibits invasion of MDA-MB-231 cells**. (A) Western blot showing knockdown of CBFβ in two independent clones. Total lysates from wild type (WT) MDA-MB-231 cells and two stable transfectants harbouring a plasmid containing shRNAs against CBFβ (shCBFβ) or a plasmid with a non-specific shRNA (shNS) were subject to western blotting analysis to determine CBFβ expression. Tubulin was used as a loading control. (B) Western blot showing expression of Runx2 in CBFβ-knockdown cells. The upper panel shows nuclear extracts from stable knockdown cells (shCBFβ) and the stable non-specific cells (shNS) after immunodetection with an anti-Runx2 antibody. The lower panel is a Lamin B1 loading control. (C) CBFβ is essential for invasion. Both CBFβ-knockdown clones and the non-specific stable clone were plated on Matrigel to determine their invasive capacity. Wild type (WT) MDA-MB-231 cells were used as positive control for invasion. Cells that migrated were stained and counted in random fields. Data are presented as mean ± standard deviation (S.D.) (n = 3). * indicates p < 0.05 compared with shNS by analysis of variance.

To confirm our findings using siRNAs we also established a stable cell line derived from MDA-MB-231 cells in which CBFβ expression was reduced using plasmids encoding short-hairpin RNAs (shRNAs). MDA-MB-231 cells were stably transfected with shRNA plasmids targeted against human CBFβ. Two independent cell lines were derived in which expression of endogenous CBFβ protein was reduced by over 80%, as determined by immunoblotting (Fig. 3A; shCBFβ, shCBFβ2). In contrast, CBFβ expression was unaffected in an independent cell line harbouring a non-specific shRNA plasmid (Fig. 3A, shNS). Runx2 protein levels were also unaffected by knockdown of CBFβ, indicating that CBFβ is not required to maintain the stability of Runx2 (Fig. 3B). Matrigel invasion assays were subsequently performed using the CBFβ knockdown cell lines. Cells with reduced CBFβ expression exhibited significantly less invasion (19%) than control cells (75%) when compared with wild-type MDA-MB-231 cells (100%) (Fig. 3C). Thus, we have demonstrated, using two independent RNA interference methods that CBFβ contributes to the invasive capacity of metastatic breast cancer cells.

### CBFβ is essential for expression of invasion genes in MDA-MB-231 cells

**Figure 4:**
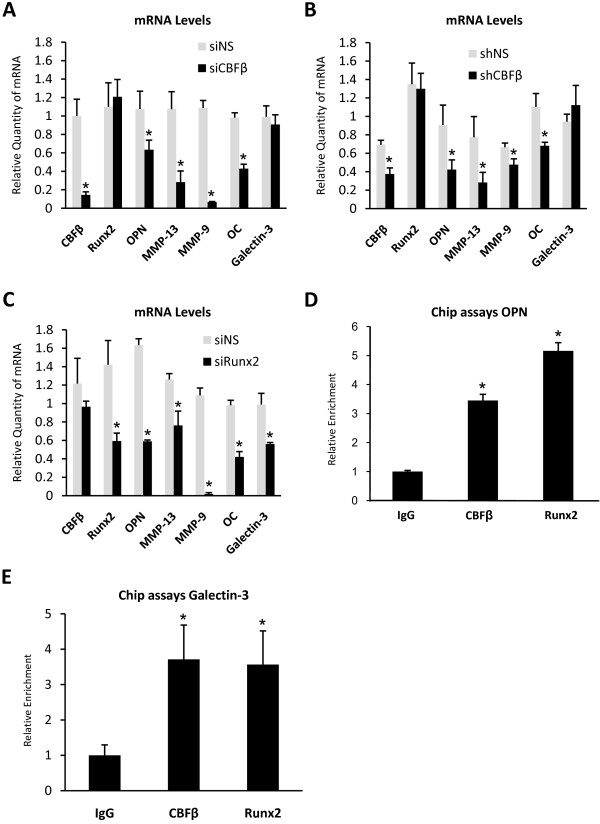
**CBFβ and Runx2 are required for expression of metastatic genes in MDA-MB-231 cells**. (A) CBFβ regulates Runx2-dependent metastatic genes in MDA-MB-231 cells. MDA-MB-231 cells transfected with siCBFβ or a non-specific siRNA were subject to real-time RT-PCR. Total RNAs were used to analyze the mRNA levels of known Runx2 target genes as indicated. Acidic ribosomal phosphoprotein P0 (RPLO) mRNA was used as a control for normalization and relative values are shown. Data are presented as mean ± standard deviation (S.D.) (n = 3). Statistical evaluation of significant differences was performed using the Student's t-test. Asterisk (*) indicates P < 0.05 when compared to control (siNS). (B) Stable knockdown cells (shCBFβ) and the non-specific cells (shNS) were subject to real-time RT-PCR. Data were analyzed as in (A) except that * indicates P < 0.05 when compared to control (shNS). (C) Runx2 regulates metastatic genes in MDA-MB-231 cells. MDA-MB-231 cells transfected with siRunx2 and a non-specific siRNA were subject to real-time RT-PCR. Data were analyzed as in (A). (D) CBFβ is recruited to the OPN promoter in MDA-MB-231 cells. ChIP assays using Runx2 and CBFβ antibodies. The relative enrichment of the OPN sequence was determined by comparing the DNA from specific antibody to that from control IgG then normalized with non-specific genomic DNA. Data are presented as mean ± standard deviation (S.D.) (n = 3). * indicates p < 0.05 compared with IgG by analysis of variance. (E) CBFβ is recruited to the Galectin-3 promoter. ChIP assays using Runx2 and CBFβ antibodies. Data were analyzed as in (D).

We next investigated the role of CBFβ in regulating the expression of a selection of Runx2-target genes. mRNA levels were measured by real-time RT-PCR in cells transfected with siRNAs targeted against CBFβ. The expression of two genes, Matrixmetalloproteinase-13 (MMP-13) and Matrixmetalloproteinase-9 (MMP-9), that are known to be involved in invasion and have been shown to be regulated by Runx2 in metastatic breast cancer cells, was examined [12,15]. We also examined the expression of two other Runx2-target genes, OPN and Galectin-3, whose expression is strongly associated with metastasis but neither has been shown to be regulated by Runx2 in metastatic breast cancer cells [16-21]. In addition, Osteocalcin (OC) expression was monitored, as this gene is known to be dependent on both Runx2 and CBFβ in osteoblasts [22]. Endogenous levels of OPN, MMP-13, MMP-9, OC and Galectin-3 mRNA were measured by real-time RT-PCR (Fig. 4A). The depletion of CBFβ resulted in a significant decrease in the expression of OPN, MMP-13, MMP-9 and OC, four of the Runx2-target genes, but no significant change in Galectin-3 expression was observed (Fig. 4A). A very similar expression profile was observed in stably transfected CBFβ-knockdown cells (Fig. 4B). We note that whilst the magnitude of some of the differences in gene expression is not as great in the stable knock-down cells, compared to the transient siRNA knockdowns, the direction of the changes and the overall trend is identical in both cases. These findings indicated that Runx2 and CBFβ are both required for expression of OPN, MMP-13, MMP-9 and OC but that Galectin-3 expression was independent of CBFβ. To establish if all five genes are indeed regulated by Runx2 in MDA-MB-231 cells, Runx2 expression was knocked down, using siRNAs. Depletion of Runx2 resulted in a significant decrease in the expression of all five genes (Fig. 4C). These data demonstrate that CBFβ is essential for full expression of OPN, MMP-13, MMP-9 and OC but not for Galectin-3. However, all five genes are dependent on Runx2, thus confirming that MMP-13, MMP-9 and OC are Runx2 targets in MDA-MB-231 cells and additionally demonstrating that OPN and Galectin-3 are Runx2 targets in these cells.

We subsequently used chromatin immunoprecipitation (ChIP) to determine whether CBFβ was selective for individual promoters by comparing the presence of CBFβ on the OPN and Galectin-3 promoters. Real-time PCR was used to amplify the OPN and Galectin-3 promoters after immmunoprecipitation of fragmented chromatin with either CBFβ-specific or Runx2-specific antibodies. Compared to non-specific IgG controls the OPN and Galectin-3 promoters were enriched approximately 3.5-fold with the CBFβ-specific antibody and 5-fold with the Runx2-specific antibody (Figs. 4D &4E). Taken together, these findings demonstrate that CBFβ is recruited to both OPN and Galectin-3 promoters but is not essential for Galectin-3 activation.

### Re-expression of CBFβ restores invasion capacity and OPN expression

**Figure 5:**
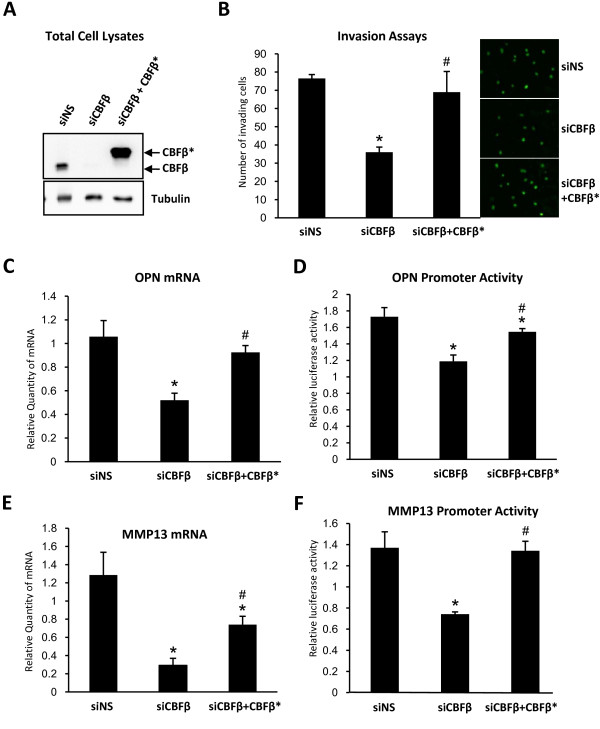
**Re-expression of CBFβ restores invasion capacity and OPN and MMP-13 expression**. (A) Western blots showing re-expression of CBFβ after siRNA knockdown. The upper panel shows total cell lysates after immunodetection with an anti-CBFβ antibody. (B) Re-expression of CBFβ restores invasive capacity. MDA-MB-231 cells were transfected with siCBFβ or siNS. siCBFβ treated cells were transfected with the Flag-CBFβ-HA expression plasmid (CBFβ*). Cells that migrated through Matrigel were stained and counted in random fields. Data are presented as mean ± standard deviation (S.D.) (n = 3). * indicates p < 0.05 compared to siNS by analysis of variance; # indicates p < 0.05 compared to siCBFβ by analysis of variance. (C) Re-expression of CBFβ restores OPN gene expression. MDA-MB-231 cells transfected with siCBFβ or siNS followed by transfection with the CBFβ* expression plasmid. Data were analyzed as in (B). (D) Re-expression of CBFβ stimulates the OPN gene promoter. MDA-MB-231 cells transfected with siCBFβ or siNS were transfected with a plasmid containing the WT OPN promoter or a mutant OPN promoter containing mutated Runx2 sites. Cells were subsequently transfected with the CBFβ* expression plasmid. Reporter activity was obtained by comparison of the WT and mutant promoter activities. Data were analyzed as in (B). (E) MMP-13 gene expression is restored by re-expression of CBFβ. The experiment was performed as described in (C). (F) Re-expression of CBFβ stimulates the MMP-13 gene promoter. The experiment was performed as described in (D) using reporter plasmids containing the WT human MMP-13 promoter or a mutant MMP-13 promoter containing mutated Runx2 sites.

We next tested the ability of CBFβ to restore the invasive capacity of MDA-MB-231 cells in which CBFβ had previously been knocked down. To prevent the re-introduced CBFβ from being knocked down by siRNAs targeted against the endogenous protein, mouse CBFβ was used. To distinguish the mouse CBFβ from the endogenous human protein in immunoblots it was also tagged with Flag and HA sequences; we refer to this protein as CBFβ*. Endogenous CBFβ was knocked down in MDA-MB-231 cells, followed by transfection with a plasmid encoding CBFβ* (Fig. 5). Cells transfected with CBFβ siRNAs and the CBFβ* plasmid expressed CBFβ* but not the endogenous CBFβ, as determined by immunoblotting (Fig. 5A). Neither form of CBFβ was observed in control transfections with siRNAs and pcDNA3, whereas endogenous CBFβ was expressed in transfections with non-specific siRNAs and pcDNA3 (Fig. 5A). Matrigel invasion assays subsequently demonstrated that the expression of CBFβ* restored the invasive capacity of the cells to similar levels observed with the non-specific siRNA controls (Fig. 5B).

To examine the effect of CBFβ* on gene expression we measured OPN and MMP-13 mRNA levels. When CBFβ* was expressed in cells transfected with CBFβ siRNAs, OPN mRNA levels were restored to similar levels observed in the non-specific siRNA transfectants (Fig. 5C). We also examined the effect of CBFβ* on the activity of the OPN promoter in CBFβ-depleted MDA-MB-231 cells (Fig. 5D). MDA-MB-231 cells transfected with siCBFβ or siNS were transfected with a plasmid containing the WT human OPN promoter or a mutant OPN promoter in which the Runx2-binding sites were mutated. The relative reporter activity was obtained by comparison of the WT promoter activity with the mutant promoter activity. In the absence of CBFβ the activity of the OPN promoter decreased by 35% compared to the non-specific siRNA controls and was restored to similar levels as the controls when CBFβ* was expressed (Fig. 5D). CBFβ* also restored MMP-13 mRNA expression and stimulated activity of the MMP-13 promoter (Figs. 5E &5F). Taken together, these data show that CBFβ is a positive regulator of metastatic-gene expression and cell invasion.

## Discussion

In this study we established that CBFβ is expressed in MDA-MB-231 cells and that it is essential for cell invasion. We subsequently demonstrated that CBFβ participates in the activation of genes implicated in cell invasion. Importantly, re-expression of CBFβ in the CBFβ-knockdown cells restored the invasive capacity. The restored invasive capacity was also accompanied by an increase in CBFβ-target gene expression, as exemplified by the increased expression of OPN. We also demonstrated that the metastatic genes OPN and Galectin-3 are Runx2-targets in metastatic breast cancer cells. However, whilst CBFβ was essential for the expression of OPN, MMP-13, MMP-9 and OC it was not required for Galectin-3 expression, despite the fact that CBFβ is recruited to the Galectin-3 promoter. CBFβ is therefore functionally redundant with respect to Galectin-3 expression in MDA-MB-231 cells. Together, our data demonstrate that CBFβ is essential for invasion of metastatic breast cancer cells and that the expression level of Runx2 is not sufficient to overcome the requirement for CBFβ, at least at a subset of Runx2-target genes.

The finding that CBFβ was present on the promoter of Galectin-3 was surprising, given that CBFβ is not required for Galectin-3 expression. One explanation for this might be that whilst CBFβ can be recruited to the Galectin-3 promoter it is not essential for Runx2 binding, possibly due to the presence of other cooperating transcription factors. Precedence for such a scenario has been demonstrated for Runx1, whereby the DNA-binding activity of Runx1 is enhanced by the cooperating transcription factor Ets-1 [23,24].

Our finding, that not all Runx2-target genes require CBFβ for their expression, is consistent with the observation that in CBFβ-deficient mice overexpression of Runx1 can only partially overcome the requirement for CBFβ. However, in MDA-MB-231 cells the genes that are dependent on CBFβ must be required for invasion since this process is inhibited in the absence of CBFβ. Presumably in the Runx1 mouse model the high expression of Runx1 is sufficient to activate a subset of genes in some cells that enables the partial rescue. The primary function of CBFβ is to enhance DNA-binding of Runx proteins and so in the absence of CBFβ, binding of Runx proteins to their target genes will be compromised. We envisage that in some cases overexpression of Runx proteins is sufficient to raise the intracellular concentration to a level that enables them to bind to a subset of target genes independently of CBFβ. Indeed, in addition to the experimental overexpression of Runx1 there are examples in nature where Runx proteins appear to function in the absence of CBFβ. In the sea urchin, CBFβ is not required for expression of the Runx-target gene PKC1, nor is it recruited to the promoter [7,8]. In the chicken, Runx1 is highly expressed in the dorsal pharyngeal region but CBFβ is not [25], and in *C. elegans *CBFβ is restricted to the hypodermis whereas the Runx protein, RNT-1, is expressed in the intestine [26]. However, it is not clear at present whether a high intracellular concentration of Runx proteins in these situations is the determining factor that enables them to bind to their target genes in the absence of CBFβ.

Perturbation of Runx2 regulatory function by mutagenesis in MDA-MB-231 cells has been shown to abolish their capacity to form osteolytic lesions in the bone, suggesting that modulating Runx2 function might be a viable means of inhibiting bone metastases [10]. Thus, our finding that CBFβ is required for Runx2 to regulate metastatic genes indicates that disruption of this interaction would also inhibit the formation of metastases. Indeed, previous work has shown that proliferation of the human leukemia cell line ME-1 can be inhibited by compounds that disrupt the interaction between Runx1 and CBFβ [27]. Such compounds may also be able to disrupt the Runx2/CBFβ interaction in metastatic breast cancer cells. The Runx2/CBFβ interaction might therefore represent a viable therapeutic target in metastatic breast cancer.

## Conclusions

CBFβ is expressed in metastatic breast cancer cells and is essential for cell invasion. CBFβ is required for expression of several Runx2-target genes known to be involved in cell invasion. However, whilst CBFβ is essential for invasion, not all Runx2-target genes require CBFβ. We conclude that CBFβ is required for a subset of Runx2-target genes that are sufficient to maintain the invasive phenotype of the cells.

## Methods

### Cell lines and cell culture

The non-metastatic MCF-7 and metastatic MDA-MB-231 human breast cancer cell lines were cultured in alpha minimal essential medium (α-MEM) containing 10% fetal bovine serum, 100 U/mL penicillin and 100 μg/mL streptomycin. Human cervical carcinoma (HeLa) cells were cultured and maintained in Dulbecco's modified Eagle's medium (DMEM) supplemented with 10% FBS, 100 U/ml penicillin and 100 μg/mL streptomycin. UMR-106 rat osteosarcoma cell line was cultured in DMEM supplemented with 10% fetal bovine serum, 1 mM sodium pyruvate, 100 U/mL penicillin and 100 μg/mL streptomycin.

### Stable CBFβ knockdown MDA-MB-231 cell line

These cell lines were established by a method described previously [28], using SureSilencing shRNA plasmids for human CBFβ (SABiosciences). Transfected cells were selected by the addition of 1 μg/mL puromycin into the media. Surviving cells were then assessed for the relative expression of CBFβ compared with the nonspecific-transfected control cell line by western blot, and the clones with the greatest degree of knockdown were selected for subsequent experiments.

### Nuclear and cytoplasmic extracts

Nuclear extracts from mammalian cells were routinely prepared as previously described [29]. The nuclear and cytoplasmic extracts were subjected to a SDS-PAGE followed by western blot.

### Immunoprecipitation and Western Blot

Whole cell extract preparations were incubated with 3 μg of Runx2 antibody (MBL) followed by incubation with Protein G agarose beads. Normal mouse IgG was used as a control. Washed beads were boiled with 50 μl of loading buffer ([0.625 M] Tris-Cl pH 6.8, 50% glycerol, 10% SDS, 10% β-mercaptoethanol, 0.05% bromophenol blue). For western blot, the samples were denatured with loading buffer and heated at 100°C prior to loading onto 12% SDS polyacrylamide gel. Proteins were detected with a mouse monoclonal anti-Runx2 (MBL), whole serum anti-Runx2 or rabbit polyclonal anti-CBFβ antibodies (Abcam). Goat anti-mouse IgG or anti-rabbit IgG conjugated with horseradish peroxidase (Pierce), were used as secondary antibodies. Immune complexes were detected by Supersignal West Dura Extended Duration Substrate (Pierce) as described previously [30].

### Luciferase constructs and plasmids

Genomic DNA from MDA-MB-231 cells was isolated following standard protocols. The human OPN promoter from -170/20 was cloned using primers 5'-GGCGGGCTCGAGGTGTGTGTGCGTTTTTGTTTTTT-3' and 5'-CCCGCCAAGCTTTGCCTCCTCCTGCTGCTGCT-3'. The 5' and 3' primers contain a XhoI and a HindIII site respectively. The amplified OPN promoter fragment was cloned into the promoter-basic luciferase reporter plasmid pGL3B (Promega). The mutant OPN promoter reporter was generated by site directed mutagenesis (Stratagene), changing the Runx2-binding site, ACCACA, to ACAGCA. The pGL3MMP-13 promoter reporter plasmid encoding the wild-type promoter sequence has been described previously [31]. pGL3MMP-13mut was generated by mutating the distal Runx2 site in the pGL3MMP-13 promoter already harbouring a mutation in the proximal Runx-binding site [31]. The subsequent plasmid has both proximal and distal Runx2-binding sites mutated. The plasmid Flag-CBFβ-HA (CBFβ*) was generated by introducing the CBFβ mouse cDNA into pcDNA3 flanked by Flag and HA tags at the N-terminus and C-terminus, respectively.

### Transient transfections and siRNAs

Cells were cultivated in 6-well plates at a concentration of 1.5 × 10^5 ^cells/well at 37°C and 5% CO_2 _atmosphere, 24 hours before transfection. Transfections were performed using lipofectamine 2000 (Invitrogen). For the luciferase assays the cells were transfected with 350 ng of the luciferase plasmids and 50 ng of Renilla luciferase plasmid and cotransfected with 400 ng of plasmid CBFβ* or pCDNA3, DNA was maintained in equal amounts. After 48 h, cells were harvested and lysates were measured for luciferase activity and normalized for transfection efficiency to Renilla luciferase activity (dual-luciferase reporter assay system, Promega). The relative reporter activity was obtained by comparison to mutant promoter activity. All readings were performed in triplicate and in three separate experiments. Data are presented as mean ± standard deviation (S.D.). MDA-MB-231 cells transfected with siRNA against CBFβ (siCBFβ), Runx2 (siRunx2), or a nonspecific siRNA (siNS) (Santa Cruz Biotechnology), were performed with oligofectamine (Invitrogen). 24 hrs after a second transfection the cells were used for mRNA extraction or invasion assays.

### Real-time RT-PCR

Total mRNA was isolated from cell pellets using an RNeasy mini kit (QIAGEN); genomic DNA was removed using the RNase-free DNase set (QIAGEN). mRNA expression of the selected genes was determined by real-time PCR using one-step QuantiTect SYBR Green RT-PCR kit (QIAGEN) according to the manufacturer's protocol. Nucleotide sequences of specific primers for the different genes were: Acidic ribosomal phosphoprotein P0 (RPLO), forward 5'-GGCGACCTGGAAGTCCAA-3' and reverse 5'-CCATCAGCACCACAGCCTT-3'; Runt-related transcription factor 2 (Runx2), forward 5'-AGCCCTCGGAGAGGTACCA-3' and reverse 5'-CGGAGCTCAGCAGAATAATTTTC-3'; Core binding factor beta (CBFβ), forward 5'-CTTAGAAAGAGAAGCAGGCAAGG-3' and reverse 5'-AACTCCAGACAGCCCATACCA-3'; Matrix metalloproteinase 13 (MMP-13), forward 5'-AAATTATGGAGGAGATGCCCATT-3' and reverse 5'-TCCTTGGAGTGGTCAAGACCTAA-3'; Osteopontin (OPN), forward 5'-TTGCAGCCTTCTCAGCCA-3' and reverse 5'-CAAAAGCAAATCACTGCAATTCT-3'; Matrix metalloproteinase 9 (MMP-9), forward 5'-ACCACAACATCACCTATTGGATC-3' and reverse 5'-ACCAAACTGGATGACGATGTCT-3'; Osteocalcin (OC), forward 5'-GTGCAGAGTCCAGCAAAGGTG -3' and reverse 5'-CAGCCAACTCGTCACAGTCC-3'; Galectin-3, forward 5'-GGGCCACTGATTGTGCCT-3' and reverse 5'-TGTTGTTCTCATTGAAGCGTGG-3'. Thermocycler conditions included an initial reverse transcription at 50°C for 30 minutes, then 95°C for 15 minutes; this was followed by a PCR program: 94°C for 30 seconds, 60°C for 45 seconds and 72°C for 45 seconds for 40 cycles. Data were collected and analysed on a Chromo4 Real-Time PCR sequence detection system (Biorad). Data are presented as mean ± standard deviation (S.D.).

### Invasion Assays

Cells were analyzed for invasion through Matrigel 24 well plates according to the manufacturer's protocol (BD Biosciences). Briefly, cells were placed in Matrigel inserts at 1.8 × 10^4 ^cells/ml in serum-free medium and were allowed to migrate for 22 hrs at 37°C. Non-migrating cells were removed from the top of the filter by scrubbing with a cotton swab. Cells that migrated were fixed and stained with DAPI (ProLong Gold, Invitrogen). Cells that migrated through the Matrigel were counted manually. Data are presented as mean ± standard deviation (S.D.).

### ChIP assays

Chromatin immunoprecipitation (ChIP) assays were performed according to the published method with modifications [32]. Briefly, MDA-MB-231 cells were cross-linked with 1% formaldehyde-phosphate-buffered saline for 10 minutes at room temperature and quenched by the addition of glycine to a final concentration of 250 mM. The cells were washed twice with cold phosphate-buffered saline and harvested. The cell pellet was resuspended in lysis buffer (50 mM Tris-Cl, pH 8.0, 10 mM EDTA, 1% sodium dodecyl sulfate). Samples were then sonicated for 15 minutes at high power with a Diagenode sonicator. After sonication, the samples were centrifuged in a benchtop microcentrifuge at 4°C, and the supernatants were diluted with ChIP dilution buffer (0.01% sodium dodecyl sulfate, 1.1% Triton X-100, 1.2 mM EDTA, 16.7 mM Tris-Cl, pH 8.0, 167 mM NaCl). For immunoprecipitation, the samples were pre-cleared using 10 μl Dynabeads protein A (Invitrogen). Four micrograms of each antibody (CBFβ or Runx2 or control IgG) was added to the sample, and the samples were incubated overnight at 4°C, followed by the addition of 10 μl Dynabeads protein A and incubation for 1 h at room temperature. The remainder of the procedure was followed according to the method described previously [32]. The DNA was isolated with Qiagen DNA purification kit. Real time PCR was performed with Osteopontin promoter primers (forward: 5'-TGAATGCCCATCCCGTAAA-3'; reverse: 5'-ATGCCCATCCCGTAAATGA-3'); 18S gene primers (forward: 5'-GTAACCCGTTGAACCCCATT-3' reverse: 5'-CCATCCAATCGGTAGTAGCG-3') and Galectin-3 as previously reported [33]. The relative enrichment of target DNA was determined by calculating the ratio of DNA from specific antibody to that from control IgG, and normalized by 18S non-specific genomic DNA within the same sample. Data are presented as mean ± standard deviation (S.D.).

### Statistical Analyses

One-way analysis of variance was used to determine statistical differences between groups using SPSS software (Chicago, IL). A *p *value < 0.05 was considered statistically significant. Multiple comparisons between individual groups were assessed by the method of Tukey. Comparisons of multiple groups to a single control were done according to the method of Dunnett. For comparison of mRNA, statistical evaluation of significant differences was performed using the Student's t-test. Asterisk (*) indicates P < 0.05 when compared to control and were considered statistically significant.

## Competing interests

The authors declare that they have no competing interests.

## Authors' contributions

DMV performed the experiments. WD performed the chip assays. CLC contributed to the discussion of the results. PS is the principal investigator and was involved in the conceptualization, discussion and writing of the manuscript. All authors read and approved the final manuscript.
